# ‘Red blood transfusion in patients undergoing cardiac surgery’

**DOI:** 10.1007/s12471-015-0696-3

**Published:** 2015-04-17

**Authors:** L. Noyez

**Affiliations:** Department of Cardio-thoracic Surgery 615, Heart Center, Radboud University Nijmegen Medical Center, PO Box 9101, 6500 HB Nijmegen, The Netherlands

To the editor,

When an author replies to the editorial comment concerning his paper, it means at least that he feels concerned [1, 2]. The comment presented three points for discussion [2].

The percentage of red blood cells (RBCs) returned to the blood bank, and the percentage of units that could no longer be used because of reduced red blood cell quality, was indeed not an endpoint of the study. However, in their introduction and in the discussion, the authors state that one of the primary rationales of the NERC protocol is to help maintenance of adequate preservation of the RBCs. With the new protocol, there are no longer unused units of blood to return to the blood bank for further use, which is an interesting point in blood conservation management. Information about the gain at this point was certainly an interesting point for discussion.

According to the protocol, RBCs are delivered to the operation room within 20 min after request. This is an important engagement, within a safety concept. However, in the same way it is supposed that trains always arrive on time, the reality is sometimes different. Therefore, it would be interesting if the protocol was evaluated on this point. In their reply, the authors claim blood was available within 20 min; however I cannot find the data proving this statement.

And the third point, that there were no catastrophic bleeding episodes in the presented series is, of course, not a reason, not to discuss how to act in case of such an unpredictable event.
